# Temporal dynamics of access to amodal representations of category-level conceptual information

**DOI:** 10.1038/s41598-018-37429-2

**Published:** 2019-01-18

**Authors:** Elisa Leonardelli, Elisa Fait, Scott L. Fairhall

**Affiliations:** Center for Mind/Brain Sciences, University of Trento, Trento, 38068 Italy

## Abstract

Categories describe semantic divisions between classes of objects and category-based models are widely used for investigation of the conceptual system. One critical issue in this endeavour is the isolation of conceptual from perceptual contributions to category-differences. An unambiguous way to address this confound is combining multiple input-modalities. To this end, we showed participants person/place stimuli using name and picture modalities. Using multivariate methods, we searched for category-sensitive neural patterns shared across input-modalities and thus independent from perceptual properties. The millisecond temporal resolution of magnetoencephalography (MEG) allowed us to consider the precise timing of conceptual access and, by confronting latencies between the two modalities (“time generalization”), how latencies of processing depends on the input-modality. Our results identified category-sensitive conceptual representations common between modalities at three stages and that conceptual access for words was delayed by about 90 msec with respect to pictures. We also show that for pictures, the first conceptual pattern of activity (shared between both words and pictures) occurs as early as 110 msec. Collectively, our results indicated that conceptual access at the category-level is a multistage process and that different delays in access across these two input-modalities determine when these representations are activated.

## Introduction

Concepts refer to a broad range of diverse notions, for example object classes (e.g.: “apple”, “face”), but also facts (e.g.: “I am Italian”, “Brad Pitt is American”). Conceptual knowledge is essential to many aspects of cognition, and understanding the neural processes that enable its representations is a central issue in cognitive neuroscience. Contemporary theories agree that the conceptual knowledge system is organised in a distributed fashion in the brain, with different theories emphasising the relative roles of ‘embodied’ representations in regions such as sensory or motor areas and representations within distributed amodal brain regions^1–12^.

A consistent finding in neuroscience is that conceptual knowledge is influenced by the category of objects to which it is referred. Strong evidence for this comes from studies on patients who exhibit category-specific semantic deficits, for example a specific inability to name or describe vegetables^13–17^. These findings have been supported by studies on healthy participants: images from different object categories reliably recruit distinct neural substrates during visual perception^18–21^. However, despite the wide use of category-based models when studying the organisation of conceptual knowledge, one critical issue regards isolating category-differences arising from the *conceptual* level of processing, from systematic differences present between categories at a *perceptual* level^5,22^. An unambiguous way to avoid the confound, is to assess categorical-differences shared by different input-modalities, thus ensuring independence from perceptual features of the stimuli.

While regions showing sensitivity to multiple object categories have reliably been demonstrated with multivariate techniques using word (or crossmodal) stimulus presentation^3,6,10,23,24^, regions exhibiting a selective response for a particular category in ventral stream are less evident and limited to sections of the parahippocampal and lateral occipital cortex^25^. An apparent exception are the input-modality independent neural substrates for people and places^22,26,27^. Across both word and picture presentation, person related concepts reliably activate the precuneus and ventromedial prefrontal cortex, while places activate the parahippocampal gyrus and retrosplenial complex.

The aim of this study is to investigate the temporal dynamics of access to conceptual knowledge, thus we recorded MEG data from participants to have a fine-grained time-resolution. We employed famous person and place as stimuli as previous fMRI studies have shown these two categories to activate different sets of brain regions at both a perceptual and semantic level^22,27^. To address the conceptual level of processing, stimuli were presented first as names, an input-modality devoid of systematic perceptual difference between categories. Successively, the same person and place exemplars were represented as pictures. We then exploited multivariate pattern analysis (MVPA), a technique that, by considering patterns of activity, enhances sensitivity with respect to traditional univariate analysis. Crucially, this strategy allowed us to isolate features that are independent from input-modality: MVPA allows generalization of the category-specific patterns of activity from names to pictures and thus the identification of conceptual semantic representations, independently from the process used to access this information. We aim to discover when conceptual knowledge common across modalities is accessed and, in particular, if this access occurs in a continuous or recursive manner. Additionally, we use words as a conceptual MVPA template to probe the emergence of amodal concepts during picture presentations. As we hypothesized that name and picture input-modalities may access conceptual representations at different times, we searched for patterns common between modalities generalizing across time, to consider possible lags between the two different input-modalities.

## Results

In each trial participants were presented with a stimulus representing either a famous person (e.g. Hillary Clinton) or a famous place (e.g. Big Ben). The experiment was designed so that in the first two runs the stimuli were depicted in the form of a written word (modality *Names*) and in the last two runs as a picture (modality *Pictures*). In each trial participants were asked to perform either a shallow categorization task (Place or Person?) or a deeper semantic task (“Italian or foreign?”).

### Behavioral analysis

We performed separate three-way repeated-measures ANOVA to assess accuracy and reaction times (RTs) across conditions (factors: task (Deep/Shallow), modality (*Names*/*Pictures*), stimulus type (Person/Place). As expected, responses were faster and more accurate in the shallow (RT: m = 676 ms std 124; acc: m = 96.3%, std 3.9%) as compared to the deep task (RT: m = 816 ms, std 108; acc: m = 86.5% std 8.5%, p < 0.001). Participants were also faster and more accurate for people (RT: m = 715 ms, std 118; acc: 92.8% std 7.5%) than places (RT: m 777 ms, std 145; acc: m 89.9% std 8.7%; p-values < 0.001). Participants were faster, but not more accurate, for *Pictures* (m = 694 ms, std 148) than *Names* (m = 798 ms, std 98, f(1,13) = 25.9 p < 0.001).

Modality by task interactions revealed that subjects were more accurate when judging pictures compared to *Names* in the shallow task and conversely when assessing *Names* compared to *Pictures* in the deep task (f(1,13) = 15.6, p = 0.002). Similarly, reaction times were faster for *Pictures* in the shallow task but did not differ between modalities in the deep task (f(1,13) = 56.7, p < 0.001). For place stimuli only, responses to *Pictures* were more accurate than to *Names* in the shallow task, while the opposite was true for the deep task (modality by task by stimulus-type interaction (f(1,13) = 25.9, p < 0.001).

### MVPA within modalities Names and Pictures

**Figure 1:**
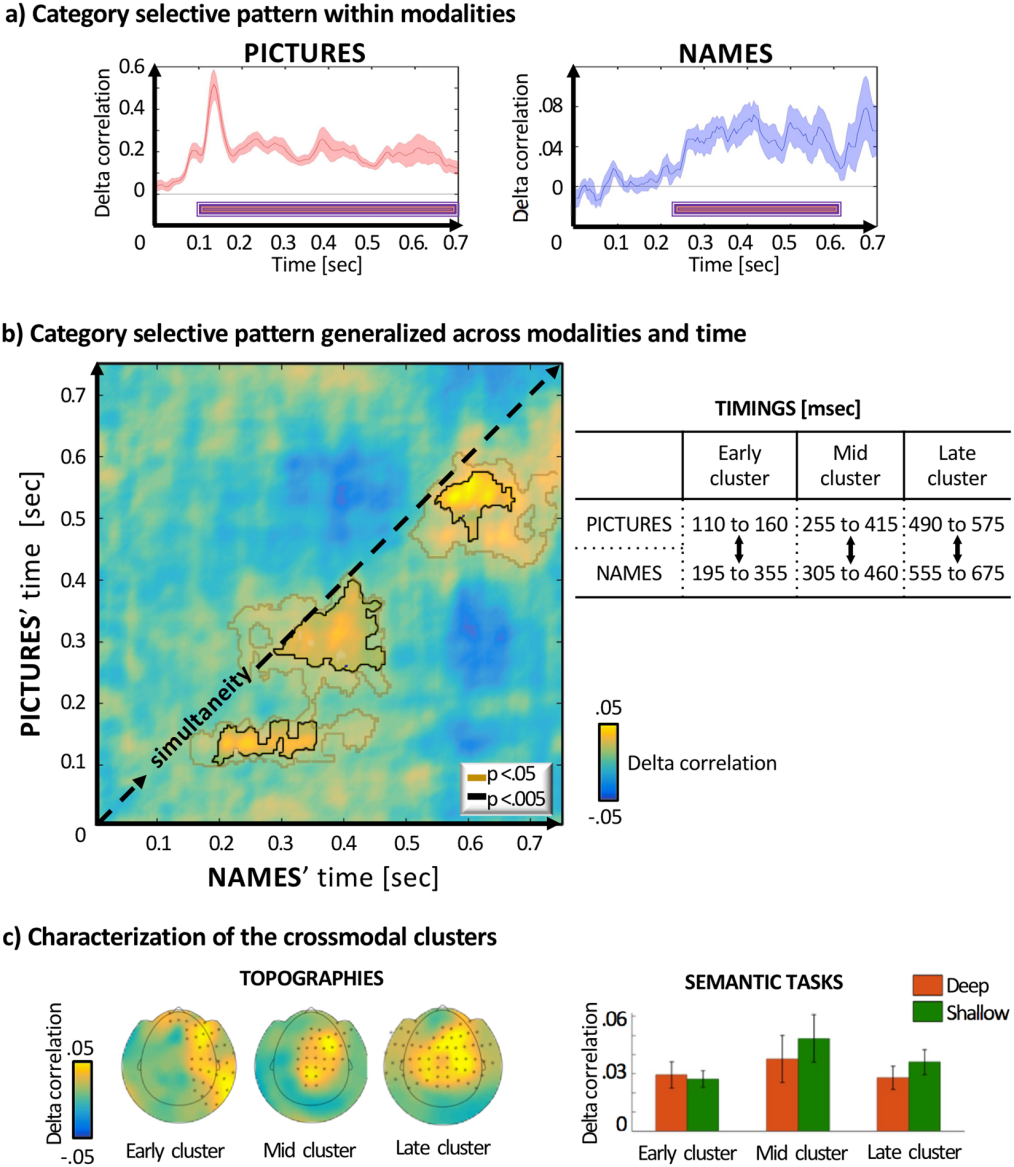
Category-specific patterns of response results from comparison of within- and between-categories. Crucially, in the cross-modal analysis within- and between-categories correlations are calculated on subsets of data across modalities. In the results shown in (**a–b-c**-left), tasks have been analyzed separately and then averaged before statistical testing. (**a**) Person/Place specific information is robustly present for both modalities (all p-values < 0.001, Montecarlo cluster-corrected). Names (230 ms to 610 ms) and Pictures (100 ms, earliest statistical point, to 750 ms). (**b**) Quantifying Places/People related information across modality revealed an early, a medium and a late cluster (all p-values < 0.005, Montecarlo cluster-corrected, orange contours initial threshold p = 0.05, black p = 0.005). Significant category-sensitive conceptual representations are off the diagonal for names, with a mean delay of 90 ms respect to pictures. (**c**) Left: Using searchlight MVPA, we revealed most informative sensors for each temporal cluster in Right: No significant differences were evident between tasks (deep/shallow) in any of these clusters.

We initially considered each modality separately and investigated if it is possible to discriminate category Persons from category Places separately for *Names* and *Pictures*. Regarding modality *Pictures*, it is possible to distinguish whether the presented stimulus is a person or a place throughout the entire epoch we analysed, i.e. the time-window 100–750 ms (p < 0.001, Montecarlo cluster-corrected, initial threshold p = 0.05, 100–750 ms). For *Names*, our analysis showed differential neural representations for Persons/Places starting at 230 and persisting until 610 ms (p < 0.001, Montecarlo cluster-corrected, initial threshold p = 0.05, 100–750 ms). In panel a) of Fig. 1 we can observe a pronounced peak at ~130 ms for *Pictures*, while words do not exhibit a similar effect.

### MVPA across modalities Names and Pictures

**Figure 2:**
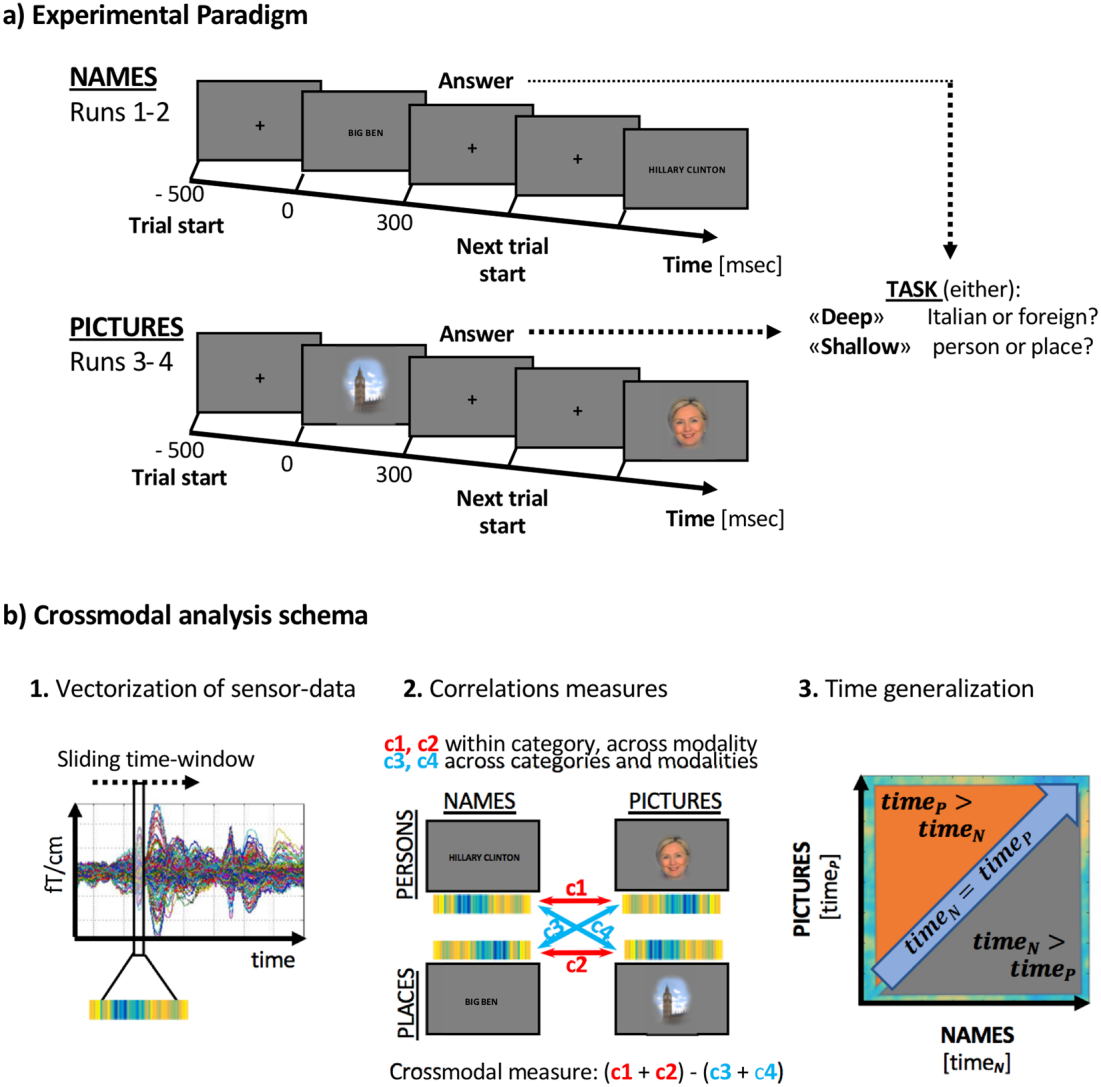
(**a**) In each trial a famous place or a famous person is presented. During the first two runs as names, then as pictures. Every block of 20 trials participants were instructed on the task they had to perform (deep or shallow semantic task). The tasks were presented in a randomly interleaved manner. (**b**) Schematic of the MVPA analysis approach, repeated for each subject, to create the Persons vs Places measure generalized across modalities and time. (1) For each condition and modality the pattern evoked across sensors is extracted at each time point (2) Category-selective patterns common across modalities are measured by comparing correlations within category but across modalities (c1/c2) and correlations across categories and across modalities (c3/c4). (3) When generalizing across time and modalities, each time point of one modality is compared with every time point of the other modality: The output is a matrix where on each axis the time of one modality is represented. When timeN = timeP (diagonal), simultaneous data points for Names and Pictures are confronted. If timeN > timeP, data points of modality names are confronted with data points occurring earlier for modality picture, meaning that in this quadrant Names are delayed with respect to Pictures. For timeN < timeP, the opposite is valid. Image of Big Ben https://www.pexels.com/photo/historical-ferris-wheel-tower-church-2212/. Image of Hillary Clinton, credit to US department of State on Visualhunt https://visualhunt.com/author/9cf212.

Our primary goal was to identify the Persons vs Places distinctive patterns that are common *across modalities*. To this end, we exploited the properties of MVPA and generalized the category-pattern found in *Names* to *Pictures*. Thereby we detected only the aspects that are common to both modalities, ensuring that our results reflect purely conceptual features. Since diverse input-modalities can lead to different latencies of activations, we performed a *time*-generalized analysis. Output of a time-generalized approach across modalities is a matrix where each axis represents the timing of a modality. In this analysis each point in time in one modality is confronted with all the other time-points of the other modality. Results that lie on the diagonal of the matrix represent the simultanoues time in both modalities. Conversely, data that lie off the diagonal represent comparisons between different time points of the two modalities, where one modality proceeds in time (or follows) the other modality (for more details on the analysis see Fig. 2, panel b)).

As shown in Fig. 1b) this analysis revealed an early, a mid and a late cluster (p = 0.006, p < 0.001, p = 0.004 Montecarlo cluster-corrected, initial threshold p = 0.005, 100–750 ms). Notably all the significant correlations are off the diagonal of our time-generalized matrix of results, revealing that category sensitive representations for *Names* are delayed with respect to *Pictures*. The first significant cluster of results across modalities for Persons vs Places occurs between 110–160 ms for *Pictures*, while for *Names* it ensues between 195–355 ms. The second cluster reveals significance in the time-window 255–415 ms for *Pictures* and 305–460 ms for *Names*. The last cluster occurs between 490–575 ms for *Pictures* and between 555–675 ms for *Names*. Thus, the initial delay of *Names* with respect to *Pictures* consists of about 140 ms and is reduced for the following clusters to about 65 ms. The approximate mean delay is 90 ms. It has to be noticed that initial time estimation is blurred due to the temporal smoothing applied to the data during MVPA analysis (±10 ms).

To gain insight into the sensors that are contributing most to cross-modal information, we run an MVPA searchlight (see Methods) across sensors-neighbours over the time windows identified in the previous section (Early cluster: *Pictures* 125–155 ms, *Names*: 210–345 ms; Mid cluster: *Pictures* 285–350 ms, *Names* 340–440 ms; Late cluster: *Pictures* 520–560 ms, *Names* 560–660 ms). As shown in Fig. 1c) This analysis indicated that information contributing to overall accuracy was coming predominantly from fronto-central sensors in all three clusters, while also being right lateralised and a more posterior source of decoding for the first cluster. We performed Montecarlo statistics within each time-window (all p < 0.005, initial threshold p = 0.05) but these values must be interpreted with caution due to partial circularity in time-window selection.

As shown in Fig. 1c), extraction of the mean values of each cluster for the two tasks separately did not reveal any statistical difference between the two tasks within each of the clusters.

### Reaction Times disparities

Analysis of the behavioural data shows that participants are significantly faster when responding to Person than Place stimuli, a result that is consistent across stimulus modality and tasks. As this difference could potentially contribute to multivariate effects, we replicated the analysis balancing RTs between conditions by selecting a subset of trials balanced RT (removing approximately 10% of trials). We performed the identical analysis as in the previous section and confirmed our previous results (all p < 0.005), thus excluding that faster reaction times for Persons than Places is the factor driving our main result.

## Discussion

In this MEG study we investigate conceptual knowledge representations shared between input-modalities names and pictures. We observed that category-specific conceptual knowledge comes online at multiple temporal stages, that category knowledge accessed via names is delayed (~90 msec) with respect to pictures and that the first amodal representation unrelated to perception (a neural pattern common to both word and picture inputs) of person/place category is accessed as early as 110 msec when viewing pictures.

We first investigated the two modalities separately. Multivariate analysis showed that when using pictures as stimuli, person/place categories are represented over an extended period of time, starting at our earliest statistical time point (100 msec) and continuing until 750 msec. Traditional univariate studies have shown that category-selective cortical activation, such as the face selective M170/N170 component, occurs at later stages^28,29^. However, recent multivariate studies have shown category selective responses reliably emerge by 100 msec^30–33^. Moreover, finely controlled univariate studies have shown face-selective effects occurring as early as 100 msec that correlate with correct categorization of a face stimulus as a face but not with its recognition^34–37^. The present results support this body of work confirming that picture-driven category effects can be reliably seen as early as 100 msec. However, within this type of stimuli it is not possible to disentangle the contribution of perceptual from conceptual category-differences (see next sections).

Regarding analysis within names, representations of object-category when concepts are cued with their written form have proved challenging to discern with electrophysiological measures^38^. Here, we observe persons/places category dissociations for word stimuli emerging at 230 msec. This result supports electrophysiological and behavioural studies that show that semantic access occurs at around 200 msec^39–41^ and extends them to show that category level semantic representations emerge in this time period.

As different input-modalities can possess different intrinsic latencies of processing, we exploited the finer temporal resolution of MEG and generalized in time: at each time point, the persons/places pattern of activation elicited by names was confronted with all the time points of pictures. This approach revealed the commonalities between representations of category elicited by words and those evoked by pictures. We observed that neural representations of category were activated both by words and pictures, with a ~90 msec delay for words, consistent with a slower access via lexical route than object-perception. Results illustrate not only that the pattern of category representation during word reading is delayed with respect to pictures but also that the two modalities converge in the brain at multiple temporal stages along neural processing.

Our crossmodal design allowed us to ask a unique and specific question. During picture viewing, robust category differences are present as early as at 100 msec. However, distinct categories of objects present systematic differences in their visual constituents, which in turn are processed by different perceptual mechanisms generating category differences that are related to perceptual characteristics. Thus, it is not possible to disentangle whether category-differences arise from perceptual or conceptual level of processing. Conversely, when the input is in the form of written names, their aspect and semantic content are independent, and it can therefore be assumed that effects are driven only by conceptual content and not perceptual. Our crossmodal time generalisation analysis found that conceptual category-specific effects are present as early as at 110 msec for pictures, while for names this occur as early as at 195 msec. In this analysis we have effectively extracted the first category selective representation evident during word presentation at 195 msec and back-projected this over the picture viewing data. We observe that the aperceptual neural representation of category emerges in the imaging data as early as 110 msec. Thus, during the natural viewing of objects in the world, we can estimate that the non-perceptual, conceptual representation of person-place categories comes online as early as 110 msec. Searchlight analysis of the information source identified a right lateralized temporal-occipital component of this cluster that is broadly consistent with face/place selective neural sources in the ventral stream and may reflect an early top-down activation of perceptual areas. This would provide support for embodied models of conceptual representation, at least at early stages^42,43^. However, further work would be needed to support this claim.

Name and picture representations converge within two later time points: an intermediate stage [names 305–460, pictures 255–415 msec] and a later stage [names 555–675, pictures 490–575 msec]. The two clusters of cross-modal activation were over centro-frontal sensors, where time and location resemble N400 and P600 respectively. Although it is tempting to speculate that the two clusters are originating from the same neural source that is reactivated, it should be noted that if this was the case, the two clusters would correlate with each other as well, in the time generalisation analysis. Instead, the results suggest that these broadly overlapping centro-frontal topographies have, in fact, different underlying neural generators.

Against expectation, the depth of semantic access had no category-selective effect on the neural response. Functional neuroimaging studies have shown that deeper semantic access preferentially activates the precuneus for person-related knowledge and TOS, PPA and RSC for place-related knowledge. It is possible that this task modulation was too subtle to be detected in the present study and that the effects we observe are driven by automatic, task-independent access to semantic representations.

At the same time, the usage of two tasks in the paradigm also addresses a potential bias: in the shallow task, response and contrast of interest are confounded (place vs person). However, this is not the case in the deep task, where responses and categories are orthogonal. If results were driven by response characteristics, this would predict statistical difference between the two tasks with a larger effect in the shallow task. As shown in Fig. 1 C-right, no evidence for such a difference is present.

As we generalized the category-selective pattern from names to pictures, our cross-modal pattern of results not only reflects semantic representations common to different input-modalities, but also ensures that conceptual processing is isolated from picture-driven perceptual confounds. Using this approach, we identified common representations of person-place categories, with a delay of approximately 90 msec from pictures to word presentation. Our results indicate that access to conceptual category-level representations is a multistage process that originates from temporal as well as frontoparietal sensors topographies. Our results show that different timings across input-modalities determine when these representations are activated. Moreover, we estimated that the first conceptual access to person-place related categorical information during vision occurs as early as 110 msec.

## Methods

### Participants

Sixteen subjects participated in this experiment (11 females; age: M = 21, SD = 2 years). All participants were right handed native Italian speakers and had normal or corrected-to-normal vision. All procedures were approved by the University of Trento Human Research Ethics Committee on the Use of Human Subjects in Research and the experiments were performed in accordance with the approved guidelines. Participants confirmed that they understood the experimental procedure and gave their written informed consent.

### Stimuli and procedures

A list of 60 famous people and 60 famous places were selected. The nationality of the famous people and places were 50% Italian and 50% foreign (persons stimuli were also balanced for gender). The same list of stimuli was presented to participants first in the written modality (*Names*) and, in the last part of the experiment, in its pictorial form (*Pictures*). The experiment consisted of four runs: in the first two runs, *Names* stimuli were presented, while in the last two runs, *Pictures* stimuli were shown. This order of presentation was chosen so that *Names* presentation was not confounded by prior exposure to particular images. Each run consisted of 12 blocks of 20 trials each (total number of trials: 960). Each block began with the instruction of the subsequent task. Participants performed a judgment task based upon either: (a) “Shallow task”, indicating the category of the stimulus (place or a person), or (b) “Deep task”, indicating the nationality of the stimulus (Italian or foreign). In each block persons and places were presented in a randomly interleaved manner. Each stimulus was presented 4 times in total in each modality. Every trial consisted of 500 ± 200 ms prestimulus interval of a fixation cross, 300 ms stimulus presentation followed by 1600 ± 200 ms presentation of a fixation cross where the answer was collected. Verbal instructions were given to each participant in the MEG room. The participants had to answer as fast and accurately as possible while pressing one of two buttons with their left or right hand.

*Names* were presented in capital letters, entirely on one row, centred around the fixation cross. No significant difference in words-length was present between People and Places (p = 0.37).

In the pictures part of the experiment, the stimuli consisted of a colour face of a famous person and place. All pictures of famous people and places had been taken from the internet. Each photo was then resized at 300 × 400 pixel, and a grey mask was applied around the face of the famous person to cover all contextual information while leaving visible their face and hair. Each image was backprojected with a VPixx PROPixx projector (VPixx technologies, Canada) at the centre of a translucent screen placed 120 cm from the eyes of the participants. The refresh rate was 120 frames per second. Timing was verified via a photodiode.

### Data acquisition

The electromagnetic brain activity was recorded using a 306 channel (204 planar gradiometers, 102 magnetometers) MEG system (Elekta-Neuromag Ltd., Helsinki, Finland) placed in a magnetically shielded room (AK3B, Vakuumschmelze, Hanau, Germany). Data was acquired with a sampling rate of 1000 Hz. Before the experiment, the individual head shape of each participant was measured using a Polhemus FASTRAK 3D digitiser (Polhemus, Vermont, USA). The procedure implied the acquisition of three fiducials points (nasion, both preauricolar sites) and the position of five coils (one on the left and right mastoid respectively, three on the forehead). Head movements were controlled for before each run of the experiment by inducing a non-invasive current through the five coils.

The dataset generated during the current study are available from the corresponding author on reasonable request.

### Data preprocessing

Offline data was visually inspected and noisy channels were excluded, prior application of MaxMove function of the Maxfilter software (http://imaging.mrc-cbu.cam.ac.uk/meg/Maxfilter) of the Elekta Maxfilter software. This function transforms the MEG data to the same head position. More specifically, all runs were aligned to the same position across all participants: using the head positions information collected throughout the scanning^44^ of all subjects, we determined a position that differed less than 2 cm from the original head-position of all subjects and all data was aligned to that position. However, for subject 1 and 13 this procedure failed and, therefore, data for those two participants was aligned only between runs within their respective runs. Subject 15’s head movements exceeded 2 cm between runs, and this subject was discarded from further analysis. Subject 14 was excluded from further analysis since their performance failed to reach the minimum level of accuracy of 75%.

Data was imported in Fieldtrip^45^ and was high-pass filtered at 0.8 Hz to remove very slow frequencies (DC offset), and at 130 Hz and filtered for line noise removal, then down-sampled to 200 Hz and each trial’s timing was corrected according to the photodiode’s information. Data was then cut into epochs of 1 s (0.2 s pre and 0.8 s post stimulus). Trials were visually inspected for possible artefacts and contaminated trials were excluded from further processing. About 4% of trials were removed from the analysis. All trials were baseline corrected, where baseline is considered the period from 200 to 40 ms prior to stimulus onset.

### Multivariate Pattern Analysis (MVPA) within and across modalities

MVPA analyses were performed using CoSMoMVPA, an MVPA toolbox in Matlab^46^ (toolbox available from http:// cosmomvpa.org).

As we were interested in investigating the general semantic mechanisms that occur commonly for different semantic tasks, the steps of the analysis described in the following sections have been conducted separately for each task, once for each modality and once across modalities. The results obtained for each task were then averaged prior to statistics.

We applied recursive correlations^47^ following a “leave-one-chunk-out method” data-folding strategy typically used in decoding studies. Trials were divided into 8 chunks, each chunk containing trials of both categories. Partitions of the data were created so that the number of trials for each category was balanced within each partition and each trial was used three times in the analysis. To determine whether the activity pattern for the category persons can be distinguished from the pattern of activation elicited by the category places, the within-category correlation, i.e. the correlation between the same category (places-places and persons-persons), was calculated for each partition. The correlations were calculated, for each partition, between the trials belonging to independent sets of trials, i.e. the two subdivisions of trials created as described in previous section. The between-category correlations, i.e. the correlation between persons and places, were calculated in the same way. The within-category correlations were then compared to the between-category correlations.

Importantly, when calculating partitions for MVPA across modalities, trials within a chunk belong to the same modality, and the two subdivisions, that are created when partitioning, belong to separate modalities. Thus, when calculating correlations, both within and between each category, these are calculated on subsets of trials across modalities.

Correlations were calculated between two sets of column vectors, where each value of the vector was the value of a certain sensor at a certain time-point. Both magnetometers and gradiometers were included in the vector, and to consider the different measurement units of magnetometer [T] and gradiometers [T/m] sensors, we applied a sensors multiplication factor to appropriately adjust for the magnitude of the signals, based on the distance of the gradiometers (17 mm). For each point in time, multivariate analysis was performed considering the pattern of activity of the central timepoint and the two adjacent samples, resulting in a multivariate analysis across a 10 msec time window. The result of each of these correlations led to a single value for each time point. The difference between within-category and between-category measures expresses a measure of the similarity-dissimilarity of the patterns for the two categories, thus leading to a single value of similarity-dissimilarity for each time point.

For the analysis across modalities we considered that different input-modalities might have different timings. We therefore used a time-generalized approach^48^, a method that extends investigation of patterns of activity by comparing (here correlating) the pattern of activity at the time-point t to the activity at the time point t’. Thus, this generalization across time implies that the neural code identified at time t recurred at time t’.

### Searchlight MVPA

As the analysis conducted in previous section across modalities revealed three significant clusters in time of common activity patterns between modalities, we were further interested in investigating from which sensors the common activity originated. For each subject we selected the data from the three time-windows where the three clusters resulting from previous analysis occurred. For each of the time windows, we averaged this data over time and then performed the same MVPA analysis across modalities described in previous section, but we used local neighbourhoods of channels in space. Neighbouring channels were considered using a template of Fieldtrip that led to an average number of neighbours of 7 for each sensor. For a given “centre” feature a sensor combination, its neighbours consisted of features for all its neighbouring sensors. Analysis were conducted separately for magnetometers and planar gradiometers and the results for the two types of sensors were then averaged for each sensor-position of the helmet. For each of the three time-windows considered, each corresponding to one of the previously identified clusters, we obtained a measure of pattern similarities for each sensor-position.

### Statistics

To statistically examine results, we separately tested the two within and across modalities results. For the within modality results, significance was tested on the time window [100 to 750 msec]. Across modality, significance was tested on the generalized time by time matrix [100 to 750 msec x 100 to 750 msec]. We performed nonparametric cluster-based dependent-samples t-tests (one-tailed) with Monte-Carlo randomization^49^, as implemented in Fieldtrip. This statistical test controls for the multiple comparisons problem (type I error rate). In particular, a t-test for each time-time pair is performed, and only t-values exceeding a certain threshold (in the case of across modalities we tested two initial-thresholds: 0.05 and 0.005) are further considered. Based on temporal adjacencies, clusters of significant differences over time are identified and are evaluated under random permutation distribution of the maximum cluster-level statistic (here 1,000 repetition). Here, only clusters showing a p < 0.0167 were considered. A similar analysis was performed for each of the searchlight analysis conducted, with the difference that clusters were evaluated on the basis on spatial adjacency instead of temporal adjacency.
